# Recreational Soccer Training Effects on Pediatric Populations Physical Fitness and Health: A Systematic Review

**DOI:** 10.3390/children9111776

**Published:** 2022-11-18

**Authors:** Filipe Manuel Clemente, Jason Moran, Rodrigo Ramirez-Campillo, Rafael Oliveira, João Brito, Ana Filipa Silva, Georgian Badicu, Gibson Praça, Hugo Sarmento

**Affiliations:** 1 Escola Superior Desporto e Lazer, Instituto Politécnico de Viana do Castelo, Rua Escola Industrial e Comercial de Nun’Álvares, 4900-347 Viana do Castelo, Portugal; 2 Instituto de Telecomunicações, Delegação da Covilhã, 1049-001 Lisboa, Portugal; 3 School of Sport, Rehabilitation and Exercise Sciences, University of Essex, Colchester CO4 3WA, UK; 4 Exercise and Rehabilitation Sciences Institute, School of Physical Therapy, Faculty of Rehabilitation Sciences, Universidad Andres Bello, Santiago 7591538, Chile; 5 The Research Centre in Sports Sciences, Health Sciences and Human Development (CIDESD), 5001-801 Vila Real, Portugal; 6 Sports Science School of Rio Maior, Polytechnic Institute of Santarém, 2040-413 Rio Maior, Portugal; 7 Life Quality Research Centre, 2040-413 Rio Maior, Portugal; 8 Research Center in Sports Performance, Recreation, Innovation and Technology (SPRINT), 4960-320 Melgaço, Portugal; 9 Department of Physical Education and Special Motricity, Faculty of Physical Education and Mountain Sports, Transilvania University of Brasov, 500068 Brasov, Romania; 10 Sports Department, Universidade Federal de Minas Gerais, Belo Horizonte 31270-901, Brazil; 11 University of Coimbra, Research Unit for Sport and Physical Activity (CIDAF), Faculty of Sport Sciences and Physical Education, 3000-248 Coimbra, Portugal

**Keywords:** football, sports medicine, physical exercise, physical fitness, physical conditioning, child, adolescent

## Abstract

This systematic review analyzed the effects of recreational soccer programs on physical fitness and health-related outcomes in youth populations. Studies were sought in the following databases: (i) PubMed, (ii) Scopus, (iii) SPORTDiscus, and (iv) Web of Science. The eligibility criteria included (1) population: youth (<18 years old) populations with no restrictions on sex or health condition; (2) intervention: exposure to a recreational soccer training program of at least four weeks duration; (3) comparator: a passive or active control group not exposed to a recreational soccer training program; (4) outcomes: physical fitness (e.g., aerobic, strength, speed, and change-of-direction) or health-related measures (e.g., body composition, blood pressure, heart rate variability, and biomarkers); (5) study design: a randomized parallel group design. The search was conducted on 6 September 2022 with no restrictions as to date or language. The risk of bias was assessed using the PEDro scale for randomized controlled studies. From a pool of 37,235 potentially relevant articles, 17 were eligible for inclusion in this review. Most of the experimental studies revealed the beneficial effects of recreational soccer for improving aerobic fitness and its benefits in terms of blood pressure and heart-rate markers. However, body composition was not significantly improved by recreational soccer. The main results revealed that recreational soccer training programs that are implemented twice a week could improve the generality of physical fitness parameters and beneficially impact cardiovascular health and biomarkers. Thus, recreational soccer meets the conditions for being included in the physical education curriculum as a good strategy for the benefit of the general health of children and young people.

## 1. Introduction

Youth populations are experiencing rising levels of obesity and declining levels of physical activity in recent times [1,2]. Moreover, epidemiological studies indicate the rise of health issues related to inactivity and sedentarism [3]. Consequently, there is growing concern over the effects of sedentary lifestyles on the health-based parameters of young people [4] as this sedentary behavior might be related to reduced quality of life and life expectancy. This issue might be explained by the different cultural and social changes experienced in recent decades, such as the increase in time spent looking at screens (TVs and video games, for example) and the development of motorized transport systems, which reduce the demand for actively physical behaviors in the youth population [5]. Hence, the World Health Organization strongly recommended in their 2020 physical activity guidelines for children and adolescents that these populations should be exposed to a minimum of 60 min/day of moderate-to-vigorous intensity physical activity. In the particular case of vigorous activities, these should be performed at least three times per week, focusing on vigorous aerobic exercise and strength-related activities that strengthen muscles and bones [6]. Based on that recommendation, programs focusing on providing youths with physical activity have recently been proposed and tested. These programs can include physical education activities, which are usually offered to children during schooltime, and recreational practices, such as soccer, which are not necessarily related to scholarly activities. In the current study, we will review the effects of recreational soccer physical activity programs on the respective pediatric populations’ physical fitness and health.

Studies have shown that the time spent in sedentary activities, defined as low-energy expenditure during waking hours, accounts for a large proportion of the day, between roughly 50 and 60% [2]. Physical activity has also been reported to reduce morbidity and mortality in adolescence [7]. Therefore, exercise—a non-sedentary behavior—might be seen as a non-pharmacological approach to decreasing the exposure to health risks for sedentary populations [5]. For example, aerobic training programs improved health parameters, such as insulin action and plasma lipids, in sedentary people [8]. Therefore, it seems that these other activities might also benefit other sedentary populations.

Different training programs could be offered to the general population when considering physical activities on a wider scale. For example, more prolonged-duration low-intensity aerobic exercises showed positive effects in sedentary subjects [3]. On the other hand, recreational sports participation—such as soccer practices—might offer higher motivation and enjoyment [9]. Indeed, a previous systematic review showed that activities with highlighted social, motivational, and competitive components were as effective and efficient as continuous running-based programs [10]. Another previous study showed that running and football-based training programs effectively improved the health-based parameters of sedentary women. However, training-induced cardiac adaptations appeared to be more consistent after football training than after running [11]. Furthermore, another study showed that recreational soccer training programs could also offer an opportunity to positively impact body composition, in comparison to people performing continuous running [12]. Hence, sports-based activities should increase adherence and improve upon the positive effects of physical activity on health parameters.

Previous studies have tested if recreational soccer practice [13,14] can positively impact physical fitness (e.g., aerobic capacity, strength, power, speed, and change of direction) health-based parameters, such as body composition (e.g., fat mass) and biomarkers (e.g., insulin) or other markers (e.g., blood pressure and heart rate variability). For example, a systematic review showed that recreational soccer increased cardiovascular and bone health and improved body composition [15]. Another study demonstrated the positive effects of recreational soccer practice on untrained women’s risk factors for bone fractures [16]. With regard to pediatric populations, it has been shown that recreational soccer can reduce risk factors associated with obesity [17] and improve different health markers [18]. Indeed, in relation to pediatric populations, adherence to physical activity programs through recreational sports practice seems even more relevant. Soccer is a popular sport worldwide and many children support clubs and players regularly. Therefore, including recreational soccer instead of generic physical activity practices could facilitate long-term adherence to physical activity and enhance the positive effects of this practice on health-related parameters in youth. Indeed, a previous study showed that greater motivation was associated with increased recreational sports participation [19]. However, to our knowledge, no previous systematic review has examined the effects of recreational soccer on health-based parameters in pediatric populations, a gap in knowledge that is addressed by the current study.

The diversity of systematic reviews in recreational soccer is clear from the extant literature [20,21,22,23,24,25,26,27]. As can be seen from the available systematic reviews, some have focused on specific outcomes, such as bone health [26,27], fat mass [22], or maximal oxygen uptake [20], while others summarized the effects on different outcomes [21,23,24,25]. Additionally, half of these systematic reviews were exclusively dedicated to adult populations [21,22,26,27], while the remaining reviews focused on mixed populations, such as children, adolescents, adults, and older individuals (both healthy and unhealthy), thus making it difficult to determine a clear overview of the effects on children and youths [20,23,24,25]. Considering the relevance of physical exercise for promoting positive physical fitness adaptations in children and young people, and the opportunities offered by recreational soccer, there is scope for a systematic review of this population. A systematic review of recreational soccer’s effects on health-related outcomes and the physical fitness of children and untrained youth populations can facilitate a precise understanding of the methodologies used in interventions and identify the adaptations induced by such interventions, in comparison to control groups who are not exposed to the same stimulus. Therefore, this systematic review aimed to analyze the effects of recreational soccer programs on physical fitness (e.g., aerobic capacity, strength, power, speed, and change of direction) and health-related markers (e.g., body composition, blood pressure, heart rate variability, and biomarkers) in untrained child and youth populations. This systematic review mainly focuses on comparisons with the control groups, aiming to understand the real effects of increased activity on health-related outcomes and physical fitness.

## 2. Materials and Methods

This systematic review followed the PRISMA 2020 guidelines [28]. The systematic review protocol was first submitted and then published on the Open Science Framework, with the registration number 10.17605/OSF.IO/FY4PX on 6 September 2022. The protocol can be accessed via the web address https://osf.io/nuebg/?view_only=11a89f39e2b34516a482953db17d2acf (accessed on 5 September 2022).

### 2.1. Eligibility Criteria

Original articles published in peer-reviewed journals or “ahead of print” were eligible for consideration. We imposed no restrictions on the language in which the gathered articles were written [29]. Table 1 presents the eligibility criteria, based on the PICOS criteria.

### 2.2. Information Sources

The following databases were searched: (i) PubMed; (ii) Scopus, (iii) SPORTDiscus, and (iv) Web of Science (core collection). The searches were performed on 6 September 2022. Additionally, manual searches were performed on the included studies’ reference lists to identify potentially relevant titles. The abstracts of these articles were checked for the relevant inclusion criteria, and, if necessary, the full text was investigated. Moreover, a consultation of two external experts (as recognized by Expertscape at the Worldwide level: https://expertscape.com/ex/soccer) (accessed on 5 September 2022) was performed, aiming to strengthen the search. Errata and article retractions were searched for each of the included articles, to identify possible sources of bias [31].

### 2.3. Search Strategy

The search was conducted using the Boolean operators AND/OR. No filters or limitations were applied to the publication date or language, to increase the chances of identifying appropriate studies [32]. The search strategy presented the following codes:

[Title/Abstract] “Soccer*” OR “Football*”

AND

[All fields/Full text] “recreation*” OR “untrain*” OR “health”

The entire search strategy can be found in Table 2.

### 2.4. Selection Process

The retrieved records (title, abstracts, and full texts) were independently screened by two of the authors (F.M.C. and H.S.). Disagreements between the two authors were discussed in a joint reanalysis. In cases where no consensus could be reached, a third author (A.F.S.) participated in a collaborative meeting to come to a final decision. The EndNote^TM^ 20.3 software (Clarivate^TM^) was used to manage the records, including the removal of duplicates, either automatically or manually.

### 2.5. Data Extraction Process

Two authors (F.M.C. and A.F.S.) independently extracted the data from the included studies. Information about study methods, results, and principal conclusions was extracted. A third author (H.S.) verified the collected information and helped in the case of any disagreements. A Microsoft^®^ Excel worksheet was designed to collect the data. If relevant information was omitted from an article, the corresponding author was contacted to help to obtain the required information. If no reply was received after establishing the first contact, we sent a second message after three days. Following that, the author was contacted twice in two days, using the same message to achieve a response or attain one two weeks after the first contact.

### 2.6. Data Items

Participant-related and context information was obtained for the following items: the date of publication, the main goal of the research; sample size; country of origin; age; sex and clinical information.

The intervention-related information included: the timing of the academic season; program duration; training frequency; level of adherence to training; dose (e.g., duration, repetitions, rest, intensity, frequency, and density); rules of play; format of play and pitch size.

The physical fitness outcomes included (but were not restricted to) cardiorespiratory-related measures (e.g., maximal oxygen uptake, maximal aerobic speed, maximal heart rate), neuromuscular-related measures (e.g., muscular power and strength), speed and change-of-direction-related measures (e.g., sprint performance, change-of-direction performance), and balance and mobility-related measures (e.g., dynamic and static balance). Health-related outcomes focused on body characteristics and body composition (e.g., body mass index, lean mass, and fat mass), blood pressure (e.g., systolic and diastolic pressure), echocardiographic measures (e.g., cardiac output), bone health (e.g., bone mineral content), biochemical parameters (e.g., total cholesterol and glucose tolerance), and inflammatory parameters (e.g., leptin).

The comparators included information about passive control groups or active control groups (namely, type of exercise, intensity, and volume).

### 2.7. Study Risk of Bias Assessment

The physiotherapy evidence database (PEDro) scale was utilized to assess the risk of bias in the included studies. This scale has previously been tested for validity and reliability [33]. The scale facilitates the rating of eleven specific study criteria, ten of which are used to classify the overall score of the article, which ranges from 0 (lowest quality) to 10 (highest quality). Usually, score thresholds provide a qualitative classification of “poor” (<4 points), “fair” (4–5 points), “good” (6–8 points), and “excellent” (9–10 points). The scale assesses the following items: C1 means that the eligibility criteria were specified; C2 means that subjects were randomly allocated to groups; C3 means that allocation was concealed; C4 means that the groups were similar at baseline regarding the most important prognostic indicators; C5 means that blinding was applied to all subjects; C6 means that there was blinding of all therapists who administered the therapy; C7 means that there was blinding of all assessors who measured at least one key outcome; C8 means that the measures of at least one key outcome were obtained from more than 85% of the subjects who were initially allocated to groups; C9 means that all subjects for whom outcome measures were available received the treatment or control condition as allocated or, where this was not the case, the data for at least one key outcome were analyzed by “intention to treat”; C10 means that the results of between-group statistical comparisons are reported for at least one key outcome; C11 means that the study provides both point measures and measures of variability for at least one key outcome. Two authors (F.M.C. and R.O.) independently reviewed and rated the included articles, based on the PEDro scale. After that, two authors (F.M.C. and R.O.) shared the scores and discussed them on a point-by-point basis. In cases where a consensus could not be reached, a third author (A.F.S.) was invited to provide their own score and make a final decision.

## 3. Results

### 3.1. Study Identification and Selection

**Figure 1:**
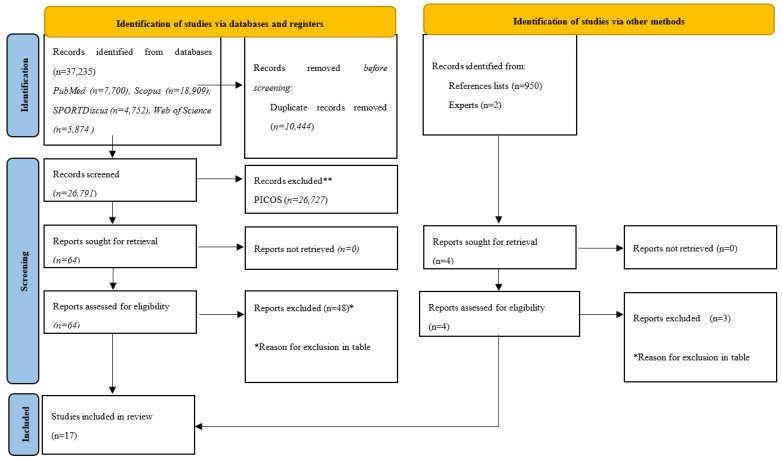
PRISMA 2020 flow diagram. * Reason for exclusion in table ** records excluded.

The initial search resulted in the identification of 37,235 titles (Figure 1). Duplicates (10,444 titles) were subsequently removed, either automatically or manually. The remaining 26,791 titles were screened for relevance, based on their titles and abstracts. Of those papers, 26,727 titles were removed. The full texts of the remaining 64 titles were then inspected, and from there, 48 more were removed, based on the eligibility criteria. Four of the potentially included articles [34,35,36,37] reported the same clinical trial registration number (NCT02000492). Based on that finding and considering that multiple reports of the same study should be collated so that each study, rather than each report, is the unit of interest in the review, as suggested by Cochrane, we chose only article [38] to remain. From the manual searches, five articles were retrieved. Of those, one was considered eligible for inclusion in the systematic review. Following the full search, 17 articles were included in the final analysis.

### 3.2. Study Characteristics

The current study’s characteristics can be found in Table 3. Among the included studies, nine were conducted in Denmark, with two performed in Brazil. Germany, China, the Faroe Islands, and Saudi Arabia yielded one study each. The remaining articles did not present any details about each study’s country of origin.

The characteristics of the recreational soccer training programs can be found in Table 4. The range of training program durations provided was between a minimum of eight weeks and a maximum of ten months. Weekly training frequency varied from two to five days. Training duration varied between 12 and 90 min. Small-sided games were used as training drills in most of the studies.

The characteristics of the control groups included in the studies can be found in Table 5. Most control groups comprised youths who were only enrolled in regular physical education classes.

### 3.3. Risk of Bias in Studies

Table 6 presents the assessment of the risk of bias. The criteria with the lowest scores were the ones associated with eligibility, the allocation being concealed, the blinding of participants and the person who administrated the protocol, and the blinding of the person who made the assessments.

### 3.4. Results of Individual Studies

Table 7 shows the main results for the physical fitness variables. The main findings showed improvements in cardiorespiratory fitness.

Table 8 shows the main results for the health variables. The main findings showed improvements in blood pressure or heart rate variables.

Table 9 shows the main results for body composition changes. Most of the studies did not find a significant impact of recreational soccer on body composition.

## 4. Discussion

The purpose of this systematic review was to analyze physical fitness and health-related markers in untrained children and youths exposed to recreational soccer (RS). The study mainly focused on comparisons with the control groups, aiming to understand the isolated effects on body composition, health-related outcomes, and physical fitness.

The main findings from this review are: (a) RS programs are appropriate for young people when an adequate and properly supervised program is followed; (b) youth RS programs spanning a period of eight to eleven weeks significantly improved cardiorespiratory fitness, blood pressure, and heart rate-related variables; (c) RS programs seem to be beneficial in improving body composition, although the results do not present a clearly discernible pattern.

### 4.1. Main Findings Regarding Physical Fitness

From the analyzed studies, the main findings showed improvements in cardiorespiratory fitness (CRF) in twelve studies. Although occurrences of cardiovascular disease (CVD) are rarely seen during childhood, the associated pathophysiological processes often begin as early as adolescence [52]. Since there is a correlation between childhood obesity and the eventual emergence of CVD risk factors in adulthood, recent studies [53,54,55] considered the importance of CVD risk factors, such as systolic blood pressure, body fat percentage, and aerobic fitness as the main outcomes related to the level of physical activity in children from 9 years of age and older. Moreover, CRF seems to prevent cardiovascular disease, regardless of body mass or composition [56,57], which highlights engagement in regular physical activity as an important behavior in controlling the aforementioned risk factors.

There is evidence from the original research in the present review that chronic exposure to RS has a high potential to improve CRF in children and adolescents with and without excess weight or obesity. RS seems to elicit high cardiovascular demands (endurance performance or VO_2_max) in healthy children and clinical populations. Moreover, this type of exercise is considered safe, with positive long-term effects on physical fitness and health indices [58].

The improvements reported by studies in terms of cardiorespiratory fitness may be as a consequence of exposure to RS, with the most effective interventions occurring two to five times per week, with intensities above ~75% HRmax, for eight weeks to ten months. Indeed, improvements in cardiorespiratory fitness for young schoolchildren have been shown to be closely related to time spent in the highest aerobic intensity zone [59].

The greater levels of daily physical activity observed in children who regularly play RS [57] may also partly explain these increases in physical fitness. In overweight and obese children, together with large improvements in terms of motor skill performance, increased exercise capacity may also facilitate greater participation in everyday activities.

Along with improvements in cardiorespiratory fitness, the studies in this review report similar improvements in 10-m [43], 20-m [40], 30-m, and 50-m [37] sprint times, increased explosive power [39], jump ability [60], agility [39], balance [36], and flexibility [36].

In contrast to the above results, Ørntoft et al. [40] report that during an 11-week period, balance and jump performance remained unchanged in both groups (IG, intervention group and CG, control group) in their study. Similarly, Larsen et al. [60] reported that ten months of soccer training did not cause significant changes in physical fitness in children. Differences in the magnitude of adaptive responses across the various studies are possibly related to the different methodologies used and the baseline values of the groups of participants in those investigations.

### 4.2. Main Findings in Relation to Body Composition

The early prevention of overweight and related diseases in children and adolescents is crucial, meaning that regular physical activity has been accepted as a means to reduce the incidence of obesity and related comorbidities [61].

Although several school-based physical activity interventions, lasting between ten and fifty-two weeks, have been reported to have had positive effects on aerobic fitness and other fitness components, few have had positive effects on body composition and body fat percentage. Some studies [39] have reported that body composition (as expressed by BMI) remained largely similar over a six-month period. In two soccer-based interventions reported by Krustrup et al. [34], in normal weight and overweight children (aged 8–12 years) who were playing small-sided soccer matches for 3 × 40 and 3 × 60 min/week for 10 and 12 weeks, respectively, there were improvements in physical fitness, but there were no changes in BMI or fat percentage. Several other studies on RS also reported that body composition variables did not change in untrained, normal-weight, overweight and obese children, and adolescents. Based on these consistent results, it is perhaps the case that short-duration intervention programs may not promote changes in body composition and that longer studies are required to observe such changes. Conversely, another study [62] observed significant decreases in BMI z-scores over similar time periods to those described herein. However, these studies also included educational and nutritional advice programs, which may have contributed to the observed decreases in BMI. It should, additionally, be noted that the use of BMI z-scores, adjusted for age and sex and using a WHO reference population, increases the sensitivity of the index [63].

Vasconcellos et al. [47] reported between- and within-group differences for body weight, BMI, and waist circumference (WC) in obese adolescents (12–17 years) who played RS only during a 12-week physical activity intervention (3 times/week; 60-min/sessions). Additionally, the intervention group showed significant decreases in body-fat percentage, while no changes were observed in the control group. Interestingly, shorter soccer-based programs (for an 11-week intervention period) such as the “FIFA 11 for Health” program, applied in several studies [40,44], and the “11 for Health in Denmark” used by Ryom et al. [50] also had positive effects on body composition, BMI (∆−0.15kg/m^2^) and fat percentage (∆−0.8%) when compared with a CG. There were within-group decreases of 23.1 ± 8.4 to 22.5 ± 8.3% in terms of body fat percentage in the intervention group, as well as an increase in lean body mass (1.0 ± 1.7 vs 0.7 ± 1.6 kg) and a lower BMI when compared to the control group.

Since changes in body fat percentage and blood pressure can be achieved through dietary manipulation, physical exercise, and other daily behaviors, an additive effect can be obtained when such methods are combined [64]. It could be speculated that there are additional effects of the “FIFA 11 for Health” program, other than the football activity itself. For example, Ørntoft et al. [40] report that education nutritional habits, when combined with high-intensity physical training, may positively influence children’s behavior. However, further studies are required to elucidate whether the increases in awareness of a healthier lifestyle that were observed following the “FIFA 11 for Health” program can result in the desired behavioral changes [65].

Another approach when analyzing the study results shows that the effects of PA on body composition parameters, such as body weight or body mass index, are inconsistent in young age groups and can distort any changes in body composition. The effects of PA on body weight are controversial, since such assessments may not consider the body composition (e.g., fat mass) [66]. The calculation of BMI uses two measures (height and weight) with a high level of variability in terms of pediatric age, which can decrease the accuracy in assessing obesity in the early stages of life.

The peri-pubertal period, in which the majority of participants in the studies herein are found, may also be a confounding factor.

Another plausible consideration is that the increase in average energy expenditure during intervention training programs can also contribute to an increase in appetite and, thus, body composition may remain constant as a result [39]. However, in most studies, nutrition was not controlled for during the intervention period and, therefore, a definitive conclusion cannot be made.

The meta-analysis conducted by Atlantis et al. [67] recommended 155–180 min of aerobic exercise per week to positively impact the fat-mass levels of overweight children. Thus, the nearly unchanged body composition reported in some studies [39,60] might be at least partly explained by an insufficient training volume (~120 min/wk). Possibly, two hours of moderate to vigorous exercise, or an energy expenditure of about 700 kcal per week, can be recommended based on the American College of Sports Medicine and the American Heart Association, aiming to maintain cardiovascular health in adults [68].

As already mentioned above regarding the study by Ørntoft et al. [40], BMI and the body-fat percentage were reduced during the 11-week period of the “FIFA 11 for Health” program. It is noteworthy that this reduction was achieved with 90 min of activity per week over an 11-week intervention period, which is much less than in previous investigations observing similar reductions in BMI and body fat percentage, as reported in interventions with higher training volume and longer duration. For example, Faude et al. [60] prescribed 180 min over 6 months, while Cvetković et al. [36] prescribed 180 min over 12 weeks, and Hadjicharalambous et al. [49] recommended 180 min/week over 8 weeks.

To allow more definite conclusions with regard to the long/short term and higher/low intensity and volume effectiveness of exercise programs in improving the body composition of normal and overweight children, further studies appear to be necessary.

However, it is important to consider that several studies [69,70,71,72] suggest that exercise improves health, even if no weight is lost, and that improved health and fitness may increase daily physical activity levels and compliance with exercise programs.

### 4.3. Main Findings Regarding Health-Related Outcomes

Of the studies in this review that analyzed the health-related outcomes of RS, two did not find any significant improvement [49,60]. The main findings showed improvements in blood pressure [34,51,73], cardiac function and HR variables [34,40], and mental and cognitive health [43,60].

Physical inactivity and lifestyle-related diseases during childhood and adolescence are associated with an increase in the risk of cardiovascular disease and largely contribute to disease and disability during adulthood. Early intervention with excessively overweight children seems mandatory for a healthier adult life [39]. This is of enormous importance to the current and future health of children and adolescents since it has been observed that obesity tends to track from childhood into adulthood [74]. Likewise, mental health is one of the current major concerns in children and adolescents [75], which may also have consequences in the future; however, in the present review, only three studies addressed this crucial issue [39].

The relationship between higher fitness levels and physical activity and the achievement of better cognitive health and performance is well established [76,77,78]. Faude et al. [60] report that the self-esteem of overweight children was considerably improved through training, with a larger effect found in the soccer-playing group. This may be explained not only by the social interactions experienced but also because of the competitive nature of the game, leading to feelings of success and coherence among the team [75]. In particular, emotional support may be enhanced when sports are conducted together with peers, an effect that is likely to be more pronounced in team sports such as soccer [79].

The study by Lind et al. [44] included an RS program (“FIFA 11 for Health for Europe”) on cognitive performance in pre-adolescent children. The authors reported a reduction in reaction time in terms of psychomotor function, attention, and working memory. Several studies have highlighted the relationship between cognitive functioning and soccer, suggesting the importance of cognitive functions for performance in this sport.

### 4.4. Behavioral Area

In the behavioral area, only the authors of [46] report that the implementation of RS in regular physical education classes seems to be a potentially appropriate stimulus for reducing aggression (physical aggression, verbal aggression, hostility, and anger) in adolescents. A recent systematic review states that the prosocial behavior of RS plays a key role in interpersonal relationships concerning the growth of children and adolescents [80].

### 4.5. Cardiovascular Adaptations

Other health-related outcomes of RS programs, such as cardiovascular adaptations, have been reported in studies showing significant structural and functional effects on the cardiovascular system. Thus, studies of normal and overweight children have reported considerable cardiovascular adaptations to medium- and short-term soccer interventions [34,38,40]. Only Hadjicharalambous et al. [49] reported that an 8-week soccer training intervention did not cause any significant changes in cardiometabolic health in adolescents.

The analyzed studies report structural and functional cardiovascular changes, such as: (a) increased left ventricular posterior wall diameter; (b) improvements in right ventricular systolic function and increased global isovolumetric relaxation time; (c) improved interventricular septum thickness, cuspid annular plane systolic excursion, and left atrial volume index and peak transmitral flow velocity in early diastole; (d) beneficial changes in endothelial function and vascular conductance; (e) decreased submaximal HR and resting and maximal HR. Such cardiac adaptations to physical exercise, known as the “athlete’s heart”, can be elicited in obese and non-obese children, who are heterogeneous in terms of fitness levels and sports participation [34]. These observations demonstrate that in childhood, the heart adapts quickly to the physiological changes induced by physical training [81]. Despite the relatively long-term duration (10 months) in the study by Larsen, Nielsen, et al. [51], short-term interventions report similar adaptations (over 10 weeks) from the application of an RS program.

It has been shown that regular and extensive training in adults is associated with changes in cardiac morphology, namely, increased left and right ventricular cavity dimension, wall thickness, and mass [82,83,84]. Cardiac adaptations to exercise also occur in children and adolescents, independent of the influence of growth and maturation [85]; a recent systematic review of echocardiographic studies concludes that these adaptations are more pronounced in structural left ventricular parameters, with the functional parameters being preserved or slightly improved by exercise [86].

However, there are still very few studies on the effects of RS training on cardiac morphology and function that use more accurate measurement methods, such as cardiac magnetic resonance or 3-D echocardiography [81].

As a consequence of the above-described cardiac adaptations to RS, in the current review, although Ørntoft et al. [40] state that there seem to be no changes in terms of resting HR, studies revealed that RS was effective in decreasing resting, submaximal, and maximal HR in normal weight, overweight, and obese children [35]. Wang et al. [37] also reported improvements in the HR index, which is determined via resting HR, exercise HR, and recovery HR (1 min post-exercise), after 30 squats in 30 s.

Autonomic heart rate regulation was evaluated via heart rate variability in two included studies [39,51]. Studies show that autonomic modulation, both at rest and during exercise, increases when a positive adaptation to training occurs, leading to an improvement in physical performance [87].

Changes that were reported in cardiac autonomic activity, namely, higher parasympathetic outflow and lower sympathetic outflow after RS programs, are concomitant with a lowered systolic blood pressure. Altogether, an improvement in vagal modulation and a reduction in sympathetic activity reflect the enhancement of hemodynamic and cardiac autonomic function in short-term programs, which can be considered a cardio-protective effect of an RS program [39,51].

### 4.6. Blood Pressure

Blood pressure (BP) is reported in some studies as showing significant reductions after the application of intervention programs, namely, the “FIFA 11 for Health” program and other RS programs [51,73]. The positive effects of physical activity programs on BP in children aged 6–12 years have been reported in a meta-analysis of physical activity intervention studies. Despite the positive effects of the interventions, there was no consensus on a reduction in all blood pressure parameters in the studies analyzed. Some studies reported a decrease only in systolic blood pressure (SBP), while others reported it only in diastolic pressure or mean blood pressure. Interestingly, some studies reported that programs of shorter duration (≤8 weeks) appear to have no effect on BP [49].

Despite the relative ambiguity of the pooled results, the beneficial effects of high levels of physical activity on blood pressure in children are clear [88,89]; even 2 mmHg reductions in systolic and diastolic blood pressure are associated with a reduction in coronary heart disease in adults [90,91,92]. It is well established that a reduction in BP, if sustained, is associated with lowered arterial stiffness and a decreased atherosclerosis progression rate in adulthood [93,94].

Thus, the present findings suggest that RS programs could be an efficient strategy for BP regulation, even in overweight and obese children, with positive health implications as one progresses through the various developmental stages to adulthood.

### 4.7. Biochemical Parameters

Besides the various tests for assessment of the cardiorespiratory system and body composition, blood biochemical parameters are a significant indicator of health status. In the present review, the studies by Vasconcellos et al. [41] and Cvetković et al. [36] report that RS promoted a statistically significant increase in the number of erythrocytes at the end of the intervention period. Both studies also verified a decrease in blood glucose. Glucose was assessed using insulin concentration, following the homeostasis model assessment of insulin resistance (HOMA-IR). The index of HOMA-IR takes under consideration both the fasting insulin values and fasting glucose values. Overweight and obese adolescents often present higher negative health outcomes than those of normal weight. This can be explained by the higher values of insulin concentration and HOMA-IR [95,96].

Concerning triglyceride blood levels, RS was shown to have favorable effects (small effect size) according to Vasconcellos et al. [41]. These results are not consistent across the different studies, perhaps due to the baseline fitness levels, the duration of interventions, and the cardiometabolic profiles of the children and adolescents [49].

It is also important to understand individual responses, due to the complexity of the participants’ levels of overweight and obesity in some studies. Studies reporting the effects of individual and group interventions on cardiometabolic risk factors in overweight or obese pubescent girls and boys are still lacking.

The completion of considerably larger and longer-term studies, including groups undergoing high and low volume and intensity soccer, will shed more light on the possible temporal developments and dose-response relationships, as well as the associated blood biochemical parameter adaptations.

### 4.8. Limitations

One of this study’s limitations is associated with the sample size, related to the included studies. Although there is no explicit information in the articles, some similar aspects were reported in papers from the same research groups that could be associated with using partial data from the same dataset (i.e., data slicing). This can artificially inflate the sample size of the current systematic review. However, since there is no reported information about that fact (in the original articles), all the included studies were considered for selection, based on the assumption of trust, and based on the fact that the articles researched different outcomes. Another limitation of this review is the limited number of subjects investigated in the studies, as well as the relatively short intervention periods, and, in this regard, the likely suboptimal statistical power. Furthermore, the studies were analyzed on a “per-protocol” basis; therefore, only the efficacy under ideal conditions can be assessed. Another limitation is that activity and caloric intake in daily living were not controlled, while seasonal influences on the investigated parameters may have occurred.

In addition, hormonal changes resulting from the onset of puberty cannot be ruled out as an influential confounding factor. Specifically, some children in the various studies were at the beginning of puberty and rapid hormonal changes may have occurred.

### 4.9. Practical Implications

Based on the current findings, it is verified that recreational soccer can effectively improve the physical fitness and health of children and young people. A two-week course of training sessions of about 60 min each, using small-sided games, can be a recommended strategy for achieving a beneficial impact on participants. However, regarding fat mass, it is recommended that this should be complemented by dietary and nutritional advice, which allows for increasing the effect since recreational soccer seems insufficient for achieving a good impact.

## 5. Conclusions

The 11-week intervention of a football-based health education program, as used in some studies, presents itself as an effective program to be used within a school’s curriculum to induce improvements in psychosocial and physiological health profiles, along with an increase in health knowledge. We recommend the tentative use of such programs alongside a call for greater efforts to engage in more studies on this particular issue. However, we would like to highlight the point that a major limitation of this review is the limited number of subjects investigated in the studies and the relatively short intervention periods, and, in this regard, the possibly suboptimal statistical power. Thus, generalizations from the findings should be made with caution.

## Figures and Tables

**Table 1:** Eligibility criteria for the current study.

	Inclusion Criteria	Exclusion Criteria
Population	Youth populations (under 18) with no restriction on sex or clinical conditions. Populations were included in Tier 0, indicating sedentary behavior, or Tier 1, indicating recreationally active, of the participant classification framework [30]; this means that: (Tier 0) they do not meet minimum activity guidelines, and, thus, can be considered inactive; or (Tier 1) they meet the World Health Organization minimum activity target and/or may participate in multiple sports/forms of activity.	Adults (>18 years old) or youths enrolled in Tiers 2 to 5 of the participant classification framework [30].
Intervention	Players were exposed to a structured recreational soccer training program for a minimum of four weeks, with no restrictions on the maximum length. Similarly, there were no restrictions on training volume, intensity, or weekly training frequency.	Exposed to less than four weeks of training intervention. Exposed to training programs for other sports than soccer.
Comparator	Passive control groups (not exposed to other training interventions, while retaining their regular physical activity levels and lifestyle) or active control groups (exposed to other exercise programs, not including recreational soccer training)	Exposed to training programs, which included recreational soccer.
Outcomes	Physical fitness outcomes (e.g., cardiorespiratory measures, speed or change-of-direction measures, muscular strength and power measures, and balance measures) and/or body characteristics or composition (e.g., body mass index, fat mass, and lean mass) and/or health-related markers (e.g., biochemical markers and inflammatory markers)	Acute physiological and/or physical responses (i.e., responses to a single training session or those experienced during exercise). Socio/psychological factors. Technical/tactical factors.
Study design	Randomized parallel group design.	No randomized designs. No controlled designs.

**Table 2:** The complete search strategy for each database.

Database	Specificities of the Databases	Search Strategy	Number of Articles in Automatic Search
PubMed	None to report	(recreation * OR untrain * OR health) AND (Soccer [Title/Abstract] OR Football [Title/Abstract])	7700
Scopus	Search for title and abstract also includes keywords	(TITLE-ABS-KEY (soccer OR football) AND ALL (recreation * OR untrain * OR health))	18,909
SPORTDiscus	None to report	TI (soccer or Football) AND TX (recreation * OR untrain * OR health)	4752
Web of Science	Search for title and abstract also includes keywords and its designated “topic”	Soccer OR Football (Title) and recreation * OR untrain * OR health (Topic)	5874

*: is the code for extension of the word.

**Table 3:** Characteristics of the included studies.

Study	Clinical Registration	Country	N	Age (Years)	Sex	Assessments (Number)	Tests Applied	Outcomes Presented in the Study
[39]	Not reported	Germany	22	10.8 ± 1.2	Both	3 (pre, mid, post)	Anthropometry; cycling ergometry; CMJ; sit-and-reach; OLS; agility run; 20-m shuttle run test	BMI; POmax; VO_2_max; lactate max; HRmax; CMJ height; change-of-direction time; 20-m shutle run time; HRmax at 20-m shutle run test; OLS time; sit-and-reach distance;
[34]	NCT01711892	Denmark	97	9.4 ± 0.4	Both	2 (pre, post)	Echocardiography; Anthropometry	BMI; systolic and diastolic BP; resting HR; LVDD; LVSD; LV; LA; LVEF; IVT; LVPWD; CO; DT; IVRT; RVDD; TAPSE
[40]	Not reported	Denmark	526	11.1 ± 0.4 and 11.0 ± 0.5	Both	2 (pre, post)	Anthropometry; X-ray absorptiometry; arterial blood pressure; 20-m sprint test; horizontal jump length; YYIRT; flamingo balance test	BMI; body fat; lean mass; systolic and diastolic BP; mean arterial pressure; resting HR; YYIRT distance; 20-m sprint time; flaming balance; horizontal jump length.
[41]	TCTR20150512001	Brazil	30	14.1 ± 1.3 and 14.8 ± 1.4	Both	2 (pre, post)	Anthropometry; X-ray absorptiometry; arterial blood pressure; cardiopulmonary exercise testing; heart rate variability; biochemical markers; endothelial function assessment; inflammatory biomarkers	BMI; body fat; fat-free mass; systolic and diastolic BP; mean blood pressure; maximal oxygen uptake; HRmax; total cholesterol; HDL; LDL; triglycerides; C-reactive protein; fasting glucose; glucose tolerance; insulin; HOMA-IR; leptin; IL-6; resistin; TNF-α; adiponectin; ET-1; NEFA.
[42]	Not reported	NA	22	15.9 ± 0.6	Boys	2 (pre, post)	Anthropometry; arterial blood pressure; heart rate variability; resting heart rate; YYIRT; 20-m sprint test; bilateral standing long jump; stork balance test.	Sum of skinfolds; systolic and diastolic BP; HR rest; *Ln SDNN; Ln rMSSD; Ln HF; HF; Ln LF; LF; LF/HF; SD1*; YYIRT distance; balance time; sprint time; jump distance.
[35]	Not reported	NA	35	11 to 13	Boys	2 (pre, post)	CMJ test; 10- and 30-m sprints; leg spreading, lying on the back; flexibility of the body when bending; flexibility of the body when stretching; biochemical markers.	CMJ; Sprint time at 10 and 30 m; leg spreading lying on the back; flexibility of the body when bending; flexibility of the body when stretching; leukocytes; erythrocytes; hemoglobin; glucose; cholesterol; triglycerides.
[36]	Not reported	NA	35	11 to 13	Boys	2 (pre, post)	Bioimpedance; CMJ test; agility *t*-test; sit-and-reach test; YYIRT; blood pressure.	BMI; body fat; lean body mass; muscle mass; CMJ; agility test; sit-and-reach test; YYIRT; resting and maximal HR; systolic and diastolic BP.
[43]	Not reported	NA	20	15.9 ± 0.6	Boys	2 (pre, post)	Anthropometry; YYIRT; 10- and 20-m sprint; sit-and-reach test; CMJ; standing long jump; stork balance test.	BMI; body fat; lean mass; sprint time; CMJ height; standing long-jump; sit-and-reach distance; balance; YYIRT distance.
[38]	NCT02000492	Denmark	295	9.3 ± 0.4	Both	2 (pre, post)	Anthropometry; X-ray absorptiometry; flamingo balance test; horizontal jump test; 20-m sprint test; coordination wall with three stages of increased difficulty.	Bone mineral content; lean mass; areal bone mineral density; the number of falls in the balance test; sprint time; coordination ability.
[44]	H-16026885	Denmark	931	11.9 ± 0.4 11.8 ± 0.2	Both	2 (pre, post)	Cognitive test battery, including detection, identification, and one-back and one-card learning tasks.	Psychomotor function; attention; working memory; visual memory.
[45]	Not reported	Faroe Islands	491	11.1 ± 0.3	Both	2 (pre, post)	Anthropometry; X-ray absorptiometry; blood pressure; stork balance test; horizontal jump test; YYIRT.	Systolic and diastolic BP; mean arterial pressure; resting HR; BMI; body fat; lean body mass; horizontal jump; postural balance; YYIRT distance.
[37]	Not reported	China	38	9 to 10	Boys	2 (pre, post)	Anthropometry; X-ray absorptiometry; 20-m shuttle run test; 50-m sprint; standing long jump; handgrip; 1-min sit up; sit-and-reach; single-leg standing,	BMI; body fat; fat mass and fat-free mass; maximal oxygen uptake; sprint time; standing long jump distance; 1-min sit-up; core muscle function; body balance; heart rate index.
[46]	No reported	NA	105	15.7 ± 0.6	Both	2 (pre, post)	Anthropometry; backward overhead medicine ball (3 kg) throw test; vertical jump test; YYIRT.	BMI; backward overhead medicine ball throw distance; vertical jump; YYIRT distance
[47]	TCTR20150512001	Brazil	13	13.9 ± 1.6 14.7 ± 2.3	Both	2 (pre, post)	Anthropometry; blood pressure; biochemical markers.	BMI; systolic and diastolic BP; body fat; HDL: triglycerides; fasting blood glucose level.
[48]	Not reported	Saudi Arabia	30	14.4 ± 2.0 15.6 ± 1.8 17.8 ± 0.4	Boys	2 (pre, post)	Blood pressure; blood glucose monitoring; biochemical markers.	LDL; HDL; triglyceride; systolic and diastolic blood pressure; total day insulin; fasting blood glucose; HbA1c.
[49]	REC-010712	NA	53	17.0 ± 0.6 16.7 ± 0.4 16.7 ± 0.4	Boys	2 (pre, post)	Anthropometry; multistage fitness test; push-up test; abdominal curl conditioning test; blood pressure.	BMI; body fat; systolic and diastolic BP; resting HR; VO2max; sit-ups; push-ups.
[50]	H-16026885	Denmark	1122	11.6 ± 0.5 11.4 ± 0.5	Both	2 (pre, post)	Anthropometry; bioimpedance; blood pressure; YYIRT; stork balance stand test; standing long jump.	YYIRT distance; VO2max; BMI; standing forward jump; balance; body fat; muscle mass; systolic and diastolic BP; resting HR.

Abbreviations: CMJ: counter-movement jump; BMI: body mass index; POmax: maximal power output; VO2max: maximal oxygen uptake; HR: heart rate; OLS: one-leg-standing; BP: blood pressure; CO, cardiac output; DT, transmitral deceleration time; IVRTglobal, global isovolumetric relaxation time; IVT, interventricular septum thickness; LA, left atrial; LVDD, left ventricular diastolic diameter; LVEF, left ventricular ejection fraction; LVPWD, left ventricular posterior wall diameter; LVSD, left ventricular systolic diameter; RVDD, right ventricular diastolic diameter; TAPSE, tricuspid annular plane systolic excursion; YYIRT: yo-yo intermittent recovery test level 1; IL-6, interleukin-6; TNF-α, tumoral necrosis factor-α; ET-1, endothelin-1; NEFA, non-esterified fatty acids; HDL, high-density lipoprotein; LDL, low-density lipoprotein; Ln, normal logarithm; SDNN, standard deviation of the normalized R–R intervals; RMSSD, root mean square of the standard deviation; HF, high frequency; LF, low frequency, SD1 geometric parameter of the Poincaré plot; LNSDNN: log-natural standard deviation of the NN (R-R) intervals; NA: not available.

**Table 4:** Characteristics of the recreational soccer training programs.

Study	Training Attendance	Duration	Days Per Week	Total Sessions	Training Duration (min)	Sets (n)	Recovery (min)	Work Duration (min)	Work Intensity	Training Drills
[39]	60 to 69%	6 months	3	54	60	NA	NA	NA	80 ± 8% HRmax	Warm-up; SSGs (50%); technique (20%); fitness courses with a ball (20%)
[34]	77 ± 18%	10 weeks	3	21 ± 5	49	NA	NA	NA	71 ± 6% HRmax	Warm-up; SSGs.
[40]	NA	11 weeks	2	22	45	NA	NA	NA	NA	Technique; SSGs
[41]	NA	12 weeks	3	36	52.1 ± 5.6	NA	NA	NA	84.5 ± 4.1% HRmax	Warm-up (10 min); SSGs (40 min; cool-down (10 min)
[42]	NA	8 weeks	2	16	NA	NA	NA	NA	NA	Warm-up; SSGs (30–45 min)
[35]	>50%	12 weeks	NA	NA	60	4	2 min	8 min per set	75.1 ± 2.3% HRmax	Warm-up (10 min); SSGs (32 min); cool-down (10 min)
[36]	>50%	12 weeks	NA	NA	60	4	2 min	8 min per set	75.1 ± 2.3% HRmax	Warm-up (10 min); SSGs (32 min); cool-down (10 min)
[43]	NA	8 weeks	2	16	30–45	NA	NA	NA	84.6 ± 6.3% HRpeak	Warm-up; SSGs (30–45 min)
[38]	NA	10 months	3	NA	NA	NA	NA	NA	0.48 ± 0.15 arbitrary units player load	Warm-up (3 to 5 min); SSGs
[44]	NA	11 weeks	2	22	45	NA	NA	NA	NA	NA
[45]	NA	11 weeks	2	22	45	NA	NA	NA	NA	SSGs.
[37]	NA	10 weeks	3	30	60	NA	NA	NA	NA	Warm-up (10 min); dribbling (10 min); dribbling and shooting (10 min); passing (10 min); running (10 min); cool-down (10 min).
[46]	>85%	32 weeks	2	64	45	4	3	5	NA	Warm-up (10 min); stretching (4 min); acceleration running (2 min); soccer (30 min); cool-down (5 min)
[47]	NA	12 weeks	3	36	60	NA	NA	NA	84.5 ± 4.1% HRmax	Warm-up (10 min); SSGs (40 min); cool-down (10 min)
[48]	21–24 n	12 weeks	2	24	90	NA	NA	NA	~80% HRmax	Warm-up (5–10 min); game
[49]	NA	8 weeks	NA	28	60	NA	NA	NA	NA	NA
[50]	NA	11 weeks	2	22	45	NA	NA	NA	NA	NA

Abbreviations: SSGs: small-sided games; NA: not available; HRmax: maximal heart rate.

**Table 5:** Characteristics of control groups.

Study	Characteristic	Duration/Frequency	Attendance	Training Intensity	
[39]	One group performing standard classes	6 months/thrice a week	72%	77 ± 6%	Warm-up; aerobic endurance activities (40%); coordination and flexibility (20%); strength (15%); speed (15%).
[34]	One group performing standard classes	10 weeks/twice a week	NA	NA	40 min of physical education classes.
[40]	One group performing standard classes	11 weeks/twice a week	NA	NA	45 min of physical education classes.
[41]	NA	NA	NA	NA	NA
[42]	Inactive group	NA	NA	NA	Kept their regular physical activity level.
[35]	One high-intensity interval training group and one control group	12 weeks/NA	NA	High-intensity interval training (80.0 ± 3.0% HRmax) Control (68.3 ± 2.2% HRmax)	Warm-up (10 min); 3 sets of high-intensity interval runs (100% maximal aerobic speed) interspaced by 3 min of passive rest; cool down (10 min) The control group performed the regular physical education classes.
[36]	One high-intensity interval training group and one control group	12 weeks/NA	NA	High-intensity interval training (80.0 ± 3.0% HRmax) Control (68.3 ± 2.2% HRmax)	Warm-up (10 min); 3 sets of high-intensity interval runs (100% maximal aerobic speed) interspaced by 3 min of passive rest; cool down (10 min) The control group performed the regular physical education classes.
[43]	Control group enrolled in regular physical education classes.	8 weeks/2 sessions a week	NA	NA	One hour of physical education classes per session.
[38]	One group performed circuit strength training and one acted as the control.	10 months/3 sessions	NA	0.34 ± 0.09 arbitrary units player load	Circuit strength training consisted of 30 s of all-out exercise with 45 s rest in between. Six to ten stations were used focusing on plyometric and dynamic or static strength (upper and lower body).
[44]	Control group enrolled in regular physical education classes.	11 weeks/2 session	NA	NA	Regular physical education classes of 45 min each.
[45]	Control group enrolled in regular physical education classes.	11 weeks/2 session	NA	NA	Regular physical education classes of 45 min each.
[37]	Inactive group	NA	NA	NA	Kept their regular physical activity level.
[46]	Control group enrolled in regular physical education classes.	32 weeks/2 sessions week	NA	NA	Regular physical education classes.
[47]	Inactive group	32 weeks	NA	NA	Kept their regular physical activity level.
[48]	Diet-only group and control group	12 weeks	NA	NA	One group had a nutritional program without exercise and the other acted as a control not receiving the program.
[49]	Control (inactive)	NA	NA	NA	Kept their regular physical activity level.
[50]	Control group enrolled in regular physical education classes.	11 weeks/2 sessions week	NA	NA	Regular physical education classes.

Abbreviations: NA: not available; HRmax: maximal heart rate.

**Table 6:** Assessment of the risk of bias.

Study	C1	C2	C3	C4	C5	C6	C7	C8	C9	C10	C11	Score
[39]	0	1	0	1	1	0	0	1	1	1	1	7
[34]	0	1	0	1	0	0	1	1	1	1	1	7
[40]	0	1	0	1	0	0	1	1	1	1	1	7
[41]	0	1	1	1	1	0	1	1	1	1	1	9
[42]	1	1	0	1	0	0	0	1	1	1	1	7
[35]	1	1	0	1	0	0	0	1	1	1	1	7
[36]	1	1	0	0	0	1	0	1	1	1	1	7
[43]	1	1	0	1	0	0	0	1	1	1	1	7
[38]	0	1	0	1	0	0	0	0	1	1	1	5
[44]	0	1	0	0	0	0	0	1	1	1	1	5
[45]	0	1	0	1	0	0	0	1	1	1	1	6
[37]	1	1	1	1	0	0	0	1	1	1	1	8
[46]	0	1	0	1	0	0	0	1	1	1	1	6
[47]	1	1	0	1	0	0	1	1	1	1	1	7
[48]	1	1	1	1	0	1	1	1	1	1	1	10
[49]	0	1	0	1	0	0	0	1	1	1	1	6
[50]	0	1	0	0	0	0	0	1	1	1	1	5

C1: eligibility criteria were specified; C2: subjects were randomly allocated to groups; C3: allocation was concealed; C4: the groups were similar at baseline regarding the most important prognostic indicators; C5: there was blinding of all subjects; C6: there was blinding of all therapists who administered the therapy; C7: there was blinding of all assessors who measured at least one key outcome; C8: measures of at least one key outcome were obtained from more than 85% of the subjects initially allocated to groups; C9: all subjects for whom outcome measures were available received the treatment or control condition as allocated, or, where this was not the case, data for at least one key outcome were analyzed according to “intention to treat”; C10: the results of between-group statistical comparisons are reported for at least one key outcome; C11: the study provides both point measures and measures of variability for at least one key outcome.

**Table 7:** Main findings of the physical fitness outcomes.

Study	Physical Fitness—Evidence of the Main Findings (Differences or Not after Training Programs in Terms of the Main Physical Fitness Outcomes)	General Effects of Soccer Training
[39]	6-month soccer training improved maximal power output, balance, flexibility, jump ability, agility, and cardiorespiratory fitness in overweight children.	Favorable
[40]	11-week soccer training improved the ability of a 20-m sprint and cardiorespiratory fitness performance in children.	Favorable
[41]	12-week soccer training increased the VO2peak in obese adolescents.	Favorable
[42]	8-week soccer training increased cardiorespiratory fitness and 20-m sprint performance in untrained adolescents.	Favorable
[35]	12-week soccer training increased explosive power and flexibility of lower extremities in overweight children.	Favorable
[36]	12-week soccer training increased agility and cardiorespiratory fitness in overweight and obese children.	Favorable
[43]	8-week soccer training decreased time in sprint performance (10 and 20 m), and increased jump ability, balance, and cardiorespiratory fitness in adolescents.	Favorable
[38]	10-month soccer training improved cardiorespiratory fitness, although without significant differences in children.	Favorable
[45]	11 weeks of “FIFA 11 for Health” improved balance and cardiorespiratory fitness in both genders. Specifically, when only girls were analyzed, cardiorespiratory fitness and jump ability were improved, while boys only improved cardiorespiratory fitness in children.	Favorable
[37]	10-week soccer training improved cardiorespiratory fitness, VO2peak, 50-m sprinting ability, jump ability, core muscle strength, and balance in children.	Favorable
[46]	8-month soccer training improved resistance in the upper body and cardiorespiratory fitness in adolescents.	Favorable
[49]	8-week soccer training improved abdominal strength and cardiorespiratory fitness in adolescents.	Favorable
[50]	An 11-week study of “11 for Health in Denmark” showed positive cardiorespiratory fitness and VO2max levels in children.	Favorable

Abbreviations:VO2max: maximal oxygen uptake; VO2peak: the peak of oxygen uptake.

**Table 8:** The main findings regarding health-related outcomes.

Study	Health-Related Outcomes—Evidence of the Main Findings (Differences or Not after Training Programs in Terms of Health-Related Outcomes)	General Effects of Soccer Training
[39]	6-month soccer training did not improve any biochemical or inflammatory marker in overweight children.	No significant effect
[34]	10-week soccer training improved the posterior wall diameter, interventricular septum thickness, and global isovolumetric relaxation in pre-adolescent children.	Favorable
[40]	11-week soccer training decreased systolic blood pressure and mean arterial blood pressure in children.	Favorable
[41]	12-week soccer training decreased systolic blood pressure, total cholesterol, triglycerides, C-reactive protein, insulin resistance, sympathetic activity, and vascular resistance. At the same time, parasympathetic activity, high-density lipoprotein cholesterol, and vascular conductance increased in obese adolescents.	Favorable
[42]	8-week soccer training increased high-frequency power, the root mean squared value of the standard deviation (rMSSD), and decreased sympathetic activity in untrained adolescents.	Favorable
[35]	12-week soccer training led to a positive effect on biochemical parameters, such as the increased number of erythrocytes in overweight children.	Favorable
[36]	12-week soccer training decreased the resting and maximal heart rate in overweight and obese children.	Favorable
[38]	10-month soccer training decreased the diastolic blood pressure and elicited discrete cardiac adaptations, such as interventricular septum thickness, cuspid annular plane systolic excursion, and left-atrial volume index in children.	Favorable
[44]	11 weeks of “FIFA 11 for Health” improved cognitive performance by reducing reaction time in terms of psychomotor function, attention, and working memory in children.	Favorable
[45]	11 weeks of “FIFA 11 for Health” decreased the systolic blood pressure in children.	Favorable
[37]	10-week soccer training improved heart function in children.	Favorable
[51]	10-month soccer training improved interventricular septum thickness and peak transmitral flow velocity in early diastole, while no other changes were observed in children.	
[47]	12-week soccer training was effective in reducing metabolic syndrome in obese adolescents.	Favorable
[46]	8-month soccer training improved the physical aggression subscale (physical aggression, verbal aggression, hostility, and anger) in adolescents.	Favorable
[48]	12-week soccer training with diet restriction decreased the glycated hemoglobin, while no other changes were shown for this group or soccer training without diet restriction, which means that diet was important to improving glycemia in adolescents with type 1 diabetes.	Favorable
[49]	8-week soccer training did not cause any significant changes in blood pressure or heart rate health in adolescents.	No significant effect

**Table 9:** Main findings of body composition.

Study	Body Composition—Evidence of the Main Findings (Differences or Not after Training Programs in Terms of Body Composition)	General Effects of Soccer Training
[39]	During the 6-month soccer training, height and weight increased in overweight children.	No significant effect
[34]	During the 10-week soccer training, body composition variables did not change in children.	No significant effect
[40]	During the 11-week soccer training, body mass index and body fat percentage decreased in children.	Favorable
[41]	During the 12-week soccer training, body mass index, waist circumference, and percentage of body fat decreased in obese adolescents.	Favorable
[42]	During the 8-week soccer training, body composition variables did not change in untrained adolescents.	No significant effect
[36]	During the 12-week soccer training, body composition variables did not change in overweight and obese children.	No significant effect
[38]	During the 10-month soccer training, body composition variables did not change in any children.	No significant effect
[45]	During the 11-week period of “FIFA 11 for Health”, height, weight, body mass index, and lean body mass increased, while body fat decreased in children of both genders.	Favorable
[37]	During the 10-week soccer training, body fat, fat mass, and abdominal fat decreased in children.	Favorable
[46]	During the 8-month soccer training, body composition variables did not change in adolescents.	No significant effect
[47]	The 12 weeks of soccer training were not enough to significantly decrease the fat percentage.	No significant effect
[49]	During 8-week soccer training, body composition variables did not change in adolescents.	No significant effect
[50]	The 11 weeks of “11 for Health in Denmark” showed a positive effect on body mass index in children.	Favorable

## Data Availability

Not applicable.
